# Improvement of Hydrogen-Resistant Gas Turbine Engine Blades: Single-Crystal Superalloy Manufacturing Technology

**DOI:** 10.3390/ma17174265

**Published:** 2024-08-28

**Authors:** Alexander I. Balitskii, Yulia H. Kvasnytska, Ljubomyr M. Ivaskevych, Katrine H. Kvasnytska, Olexiy A. Balitskii, Radoslaw M. Miskiewicz, Volodymyr O. Noha, Zhanna V. Parkhomchuk, Valentyn I. Veis, Jakub Maciej Dowejko

**Affiliations:** 1 Department of Strength of the Materials and Structures in Hydrogen-Containing Environments, Karpenko Physico-Mechanical Institute, National Academy of Sciences of Ukraine, 79-601 Lviv, Ukraine; ivaskevich@ipm.lviv.ua; 2 Department of Mechanical Engineering and Mechatronics, West Pomeranian University of Technology in Szczecin, 70-310 Szczecin, Poland; 3 Department of Physico-Chemistry of Casting Processes, Physico-Technological Institute of Metals and Alloys NAS of Ukraine, 03-142 Kyiv, Ukraine; kvasnytska@ptima.kiev.ua (Y.H.K.); katonish@gmail.com (K.H.K.); volodymyrnoha99@gmail.com (V.O.N.); zhanna_parkhomchuk@ukr.net (Z.V.P.); valentynveis@gmail.com (V.I.V.); 4 Department of Chemistry, University of Waterloo, Waterloo, ON N2L 3G1, Canada; olexiybal@yahoo.com; 5 Research Center for Management of Energy Sector, Institute of Management, University of Szczecin, 71-004 Szczecin, Poland; radoslaw.miskiewicz@usz.edu.pl (R.M.M.); jakub.dowejko@usz.edu.pl (J.M.D.)

**Keywords:** hydrogen-resistant superalloy, directional crystallization, water-based binder, gas turbine engine blade, ceramic mold

## Abstract

This paper presents the results of an analysis of resistance to hydrogen embrittlement and offers solutions and technologies for manufacturing castings of components for critical applications, such as blades for gas turbine engines (GTEs). The values of the technological parameters for directional crystallization (DC) are determined, allowing the production of castings with a regular dendritic structure of the crystallization front in the range of 10 to 12 mm/min and a temperature gradient at the crystallization front in the range of 165–175 °C/cm. The technological process of making GTE blades has been improved by using a scheme for obtaining disposable models of complex profile castings with the use of 3D printing for the manufacture of ceramic molds. The ceramic mold is obtained through an environmentally friendly technology using water-based binders. Short-term tensile testing of the samples in gaseous hydrogen revealed high hydrogen resistance of the CM-88 alloy produced by directed crystallization technology: the relative elongation in hydrogen at a pressure of 30 MPa increased from 2% for the commercial alloy to 8% for the experimental single-crystal alloy.

## 1. Introduction

To meet the constantly growing demands for the quality and operational reliability of modern engines, the production of new GTEs requires improved technological processes and development, as well as the implementation of high-quality new methods and processing techniques. The continuous connection between the engine’s design and the technology of its production motivates the creation of new casting technologies to obtain complex-profile castings of high quality. The primary necessity in contemporary industry is to enhance efficiency by increasing the turbine’s gas inlet temperature.

The most critical components of a GTE are the working and nozzle blades of the turbine. In recent years, the production of marine and power gas turbine engines has been actively focused on improving their operational characteristics, including both mechanical strength and corrosion resistance. This improvement has been achieved through the utilization of special heat-resistant alloys containing refractory metals, such as rhenium, tantalum, and ruthenium. These alloys contribute to the increased resistance of the blades to high temperatures and aggressive environments, significantly improving the performance and service life of the GTE and ultimately fostering the development of industry [1,2,3,4,5,6,7].

At the Physico-Technological Institute of Metals and Alloys (PTIMA), in collaboration with the Karpenko Physico-Mechanical Institute (PMI) of the National Academy of Sciences of Ukraine and domestic gas turbine manufacturing enterprises, research is being conducted on existing alloy grades (and the development of new ones) that use alloying refractory metals (Mo, W, Nb, Ta, Re) and on their complex influence on the properties of heat-resistant corrosion-resistant nickel-based alloys. During the research, the reduction of chromium content to 12.0%…13.2% by mass was successfully achieved to enhance hydrogen resistance without compromising the corrosion resistance of the alloy. The phase–structural stability and strength characteristics of the alloy were preserved according to the certification requirements for turbine blades, ensuring a prolonged operational lifespan within the range of 25,000–30,000 h. These blades are utilized in combustion products of hydrogen-containing diesel fuel and natural gas [8,9,10].

Alongside the development of new compositions of heat-resistant alloys for GTE blades, one of the ways to enhance the operational properties of these components is through the method of their melting, including single-crystal growth technology. In the production of gas turbines at manufacturing plants, cast turbine blades are formed with directional or single-crystal structures, depending on the level of responsibility of their intended use. It is known that excluding transverse grain boundaries in the blade structure through DC of the melt in a ceramic shell mold allows for improved operational properties of the turbine blade (heat resistance, thermal stability, and plasticity) [1,11,12,13,14,15,16,17].

Modern Single-Crystal Superalloy Manufacturing Technologies. Among the recent technologies in the casting of turbine blade blanks, the process of high-gradient DC has been introduced. In this process, the directed dendritic structure in alloys is formed at the solid–liquid interface in the temperature range between solidus (T_S_) and liquidus (T_L_) temperatures. The length of the mushy zone is determined by measuring the temperature gradient at the growth front. This gradient indicates how rapidly the temperature changes along the directed movement of the metal crystallization front. In the presence of a low temperature gradient on the casting surface, the phenomenon of “stream-like banding” may also occur [1]. This manifests as chains of equiaxed grains oriented in the direction of crystallization and consists of excess eutectic phase segregations in heat-resistant alloys. This structure arises under specific cooling and crystallization conditions and influences the material’s properties [1,18].

The Bridgman–Stockbarger method (high-rate solidification (HRS)) [19,20,21,22,23,24,25,26,27,28,29,30,31,32,33,34,35,36,37] is widely used in global practice and at enterprises for the production of GTE blades with a directional structure at the initial stages of implementing the casting process. This process involves pouring a heat-resistant melt into a ceramic mold that is placed on a copper cooling crystallizer. The crystallizer, positioned vertically, moves into the cooling zone at a specified speed during melting, resulting in temperature control at the crystallization front. Intensive heat dissipation occurs in the lower part of the mold through the copper crystallizer. Simultaneously, the upper part of the mold cools through radial heat dissipation and heat radiation from the lateral surface of the mold to the chamber walls. This process leads to DC.

Another effective method commonly used in gas turbine manufacturing for heat dissipation is the DC process with liquid metal cooling—the liquid metal cooling (LMC) method [11,20,21,22,23]. The distinct feature of this process is that the mold with a heat-resistant melt is vertically moved from the heating chamber to the cooling chamber at a controlled speed into a vessel with a cooling melt (aluminum, tin). The use of liquid metal coolant enhances the heat dissipation rate. However, the growth of this cooling method presents several technological challenges related to regulating the temperature of the ceramic mold with the melt.

Companies that manufacture GTEs to obtain blades without cooling cavities successfully utilize the gas cooling casting (GCC) method with gas jet cooling in a vacuum [24,25]. The DC process using GCC involves cooling ceramic molds with a directed argon jet during the crystallization process. The cooling effect on the molds is maintained through radiation. This method allows for optimal temperature control during casting, ensuring high quality and an excellent crystalline structure for large blades. The use of GCC is a crucial aspect in the production of high-precision components for gas turbine engines.

Many researchers over several decades have focused their efforts on the investigation, calculations, and development of control methods in the DC process [26,27,28,29,30,31,32,33]. In this context, key tasks for thermal calculations include determining the optimal crystallization speed of blades, the temperature gradient at the crystallization front, the cooling speed of molds, the extent of the solid–liquid transition zone of the alloy, and its positioning relative to the cooling or heating level. These parameters are crucial for achieving optimal conditions in the DC process and improving the characteristics of the final product.

To achieve a consistently oriented structure in the DC process, it is important to create conditions in which the crystal growth rate is maximized while the nucleation rate is minimized. This allows for the desired alignment of the crystalline structure and enhances the quality of the final product. Therefore, the first objective of this study is to investigate the influence of temperature–speed conditions in directional growth on the formation characteristics of the oriented dendritic-cellular structure in high-speed casting units for obtaining high-precision cast GTE blades.

The development or improvement of new GTE designs requires the manufacturing of experimental batches of blades, the acquisition time for which currently ranges from six months to one year, attributed to the prolonged process of equipment fabrication. An effective solution to this problem could be the application of additive technologies in the production of disposable models of blades.

In the industry, a suspension of ethyl silicate is commonly used as a bonding agent; however, its production requires the use of organic solvents that are explosive and flammable, such as acetone or ethyl alcohol. In the production of ceramic shell molds based on ethyl silicate, the evaporation of alcohol from tanks containing the suspension and drying shells requires the installation of equipment for the collection and reduction of emissions of harmful substances. Precautionary measures should also be followed during the transportation and storage of alcohol suspensions. It is necessary to implement a system for the continuous and precise control of temperature and humidity in drying compartments, as the suspension can transform into a gel. In this case, it should be disposed of, along with other production waste. Production methods of shell molds based on a water-based system eliminate these environmental and transportation drawbacks. Previous studies have indicated that tested samples, which are entirely based on a water system with the addition of Remasol, exhibit a higher level of open porosity in the raw state after heating to 950 °C and after firing. Specifically, the open porosity increases from 7.51% to 12.23%, which is 5.62% higher than in samples using a combined alcohol system or based on ethyl silicate [34,35].

A simpler process for preparing a suspension involves adding a filler to the bonding material and stirring until the required viscosity is achieved. Shells dried on a water-based binder are less sensitive to drying conditions, allowing for the production of strong shells, even at high humidities and temperatures. In the case of rapid solvent evaporation, an ethyl silicate shell mold may become brittle and crack. There is no need to dispose of a previously prepared suspension during work breaks, as it has a longer shelf life, which can be up to six months when stored under appropriate conditions. The use of a water-based binder in the technology improves the surface quality of the molds and helps eliminate problems with undercuts due to the better “breathing” of the shells. Fire safety issues and concerns related to emissions cleanup are addressed because water-based binders do not contain solvents or volatile substances.

To obtain high-quality shell molds from heat-resistant corrosion-resistant alloys using the directional crystallization method, the manufacturing process of ceramic shell molds should be improved, taking into account modern environmentally friendly technologies, which is the second objective of this work.

## 2. Materials and Methods for Investigation

**Figure 1:**
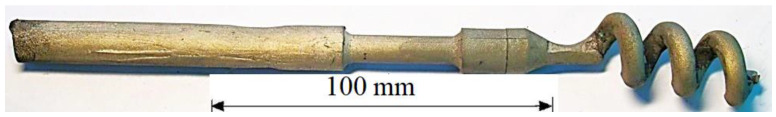
External view of the experimental sample.

The experiments were conducted to establish optimal conditions for DC and improve the technological process of obtaining a regularly oriented structure in the castings of the second-stage turbine blades of the UGT 5000 gas turbine engine (engine power 5000 MW, manufactured by Gas Turbine Research & Production Complex “Zorya”-“Mashproekt”, Mykolaiv, Ukraine). The material for the experimental melts was the heat-resistant alloy CM88 [2,9,33], whose chemical composition is shown in Table 1. The research was carried out on melted standard samples of the alloy (Figure 1). The determination of the quantity of main components of the alloys and impurities was performed by the chemical method using standard techniques, and microalloying additives were controlled by the chemical-spectral method with a relative error of ±0.001%. 

The microstructure of the samples was examined using the Tescan Mira 3 LMU scanning electron microscope (Brno, Czechia), with the following main characteristics: spatial resolution: 1 nm@30 kV, 2 nm@3 kV; accelerating voltage: 200 V–30 kV; working pressure in the chamber: high vacuum mode ≈ 9 × 10^−3^ Pa; low vacuum mode 7–150 Pa.

The technological process developed for obtaining prototype blades for gas turbines (GTs) involved the use of 3D printing. The production of prototype models from polylactic acid (PLA) was carried out on a Klema 3D printer with a layer height of 0.2 mm, according to the provided drawings.

The ceramic mold was produced using the traditional investment casting technology by melting models, involving the immersion of the model block in a suspension, followed by coating it with electrocorundum. The thickness of the ceramic mold was 10–12 mm. A suspension without coating was applied as a surface layer to create a smooth mold surface.

For the sake of environmental friendliness in the experiments, the water-based binder Remasol (silica sol) [34] was used. The preparation of the suspension based on the water-based binder was carried out by mixing its components without cooling using a mixer with an impeller rotation frequency of 1000–2800 rpm.

The introduced polymer accelerates the drying of water-based binders. The drying temperature range is as follows: the front layer—3–4 h at a temperature of 21–30 °C. Drying of subsequent layers occurs under the same parameters but with intensive air blowing at a speed of 1–3 m/s. Industrial fans of any design can be used. In both cases, it is important to maintain the relative humidity of the air at 40–60%. In this case, the drying of subsequent layers will take 1 h per layer. Increasing the relative humidity leads to an extension of the drying time but does not affect the shell parameters, provided that the drying is fully completed. Sintering is carried out at a temperature of 900–950 °C without a supporting filler for 4–6 h. After this, it is necessary to pour metal directly into the hot shell.

**Figure 2:**
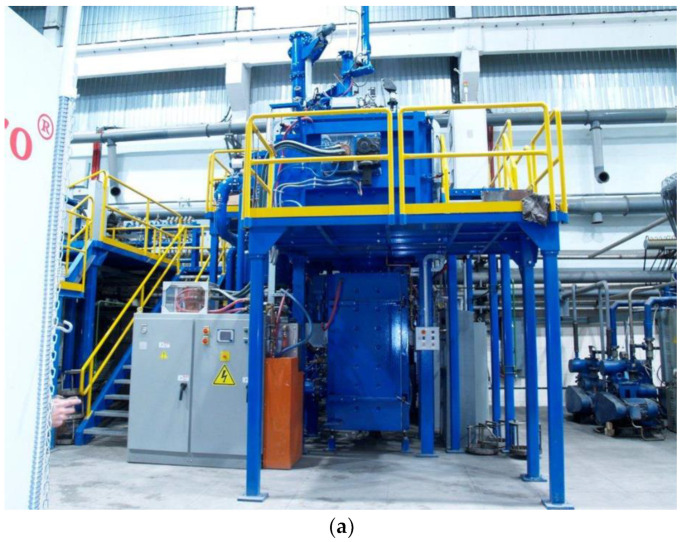

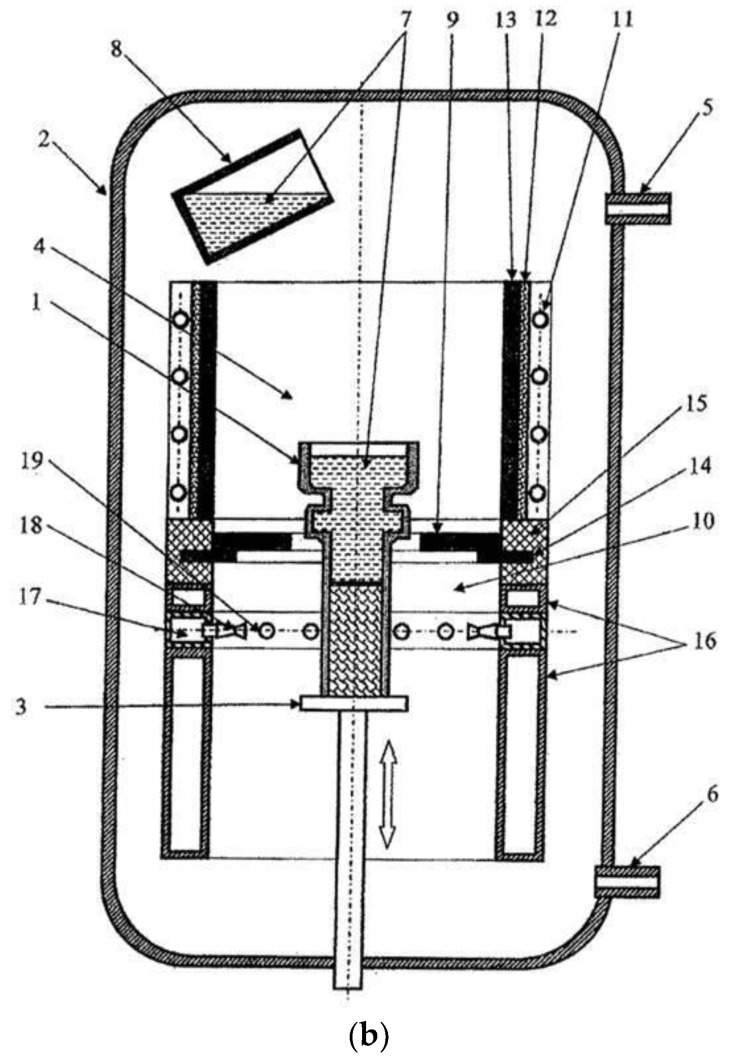
Exterior (**a**) and scheme (**b**) of the casting setup with an additional cooling unit: 1—casting mold; 2—vacuum jacket (vacuum chamber); 3—crystallizer; 4—heating zone of the technological chamber; 5—connection pipe for the system; 6—connection pipe for connecting to the additional unloading system of the cooling zone of the working chamber; 7—molten metal; 8—crucible of the loading device with molten metal; 9—thermal insulation screen with a central hole; 10—cooling zone of the technological chamber; 11—inductors of the heating zone; 12—thermal insulation of the inductor; 13—graphite muffle furnace; 14—stopper ring of the thermal insulation screen; 15—cylindrical wall of the thermal insulation screen; 16—jacket of the technological chamber in the cooling zone; 17—ring gas collector; 18—gas ejectors; 19—holes for gas ejectors [34].

The melting and pouring were carried out in the vertical-type vacuum furnace VIM-25-175C (manufacturer: “SECO-WARWICK,” Świebodzin, Poland), in which the crystallization process was implemented according to the GCC method (Figure 2) [37]. The crucible capacity for melting metal in this setup was 15 kg. Argon gas with a purity of 99.98% from 50-L cylinders at a pressure of 200 bar was used for gas cooling of the molds with molten metal. Supersonic nozzles with a Mach number of 2.8 and a critical diameter of 0.7…1.5 mm were used to achieve a high temperature gradient. These nozzles were assembled in a ring collector, providing the ability to change the direction of the cooling gas supply. They were positioned adjacent at a distance of 45 to 75 mm from the lower surface of the thermal insulation screen. The angle of inclination of the nozzle axis downward from the horizontal plane was set at 20°. The application of such a cooling method enhances the crystallization process of the molten metal by changing the temperature gradient at the crystallization front compared to convective cooling in a vacuum. This is achieved by the presence of a copper tray or by immersing the mold in the liquid metal coolant. This process significantly influences the kinetics of material structure formation and improves its properties [38,39,40].

**Figure 3:**
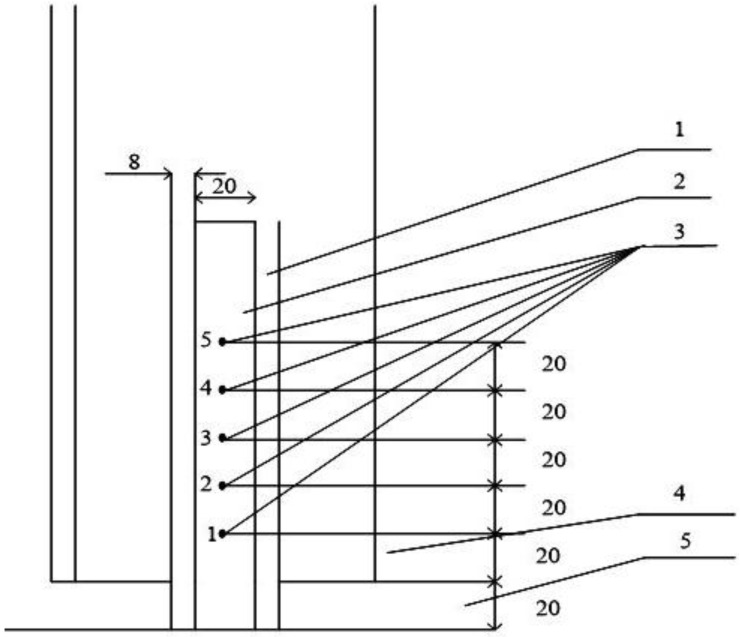
Scheme of thermocouple arrangement: 1—ceramic mold; 2—metal sample; 3—thermocouples; 4—graphite crucible; 5—ceramic substrate; mm.

Local values of directed crystallization process parameters during melting were calculated based on the temperature distribution in the volume of the mold, which was experimentally measured at five points along the axis of the mold using tungsten–rhenium thermocouples (type WR 5/20, electrode diameter 0.30 mm) (Figure 3). According to the single-crystal technology for obtaining turbine blades with a directed structure with crystallographic orientation [001], for each sample, a crystal initiation seeds from the Ni–65% mass. W–35% mass alloy was placed in the crystal nucleation zone at the bottom of the ceramic mold. The time required to achieve the working vacuum at the level of 7 × 10^−2^ Pa in the chamber before the start of the casting process after loading the raw materials was 2 min.

Mechanical properties were determined on five-fold cylindrical specimens with a working part diameter of 5 mm. Tests in hydrogen at a pressure of 30 MPa were carried out after two washings with hydrogen and intermediate vacuuming of the working chamber to a pressure of 10^−3^ Pa. The volume fraction of hydrogen in the working chamber reached 99.9997%, the volume fraction of oxygen did not exceed 0.00007%, and the mass concentration of water vapor was 0.0009 g/m^3^.

## 3. Results and Discussion

Experimental studies were conducted to establish the technological parameters of the DC process to obtain a regular dendritic structure for castings. The temperature of pouring the melt into the mold was 1580 °C, and the temperature of the ceramic mold with the melt in the nucleation zone was 1430 °C. Upon visual inspection of the external and internal surfaces of the castings made of the nickel-based alloy and metallographic examinations, no micropores, defects, shells, or nonmetallic inclusions were detected after pouring into molds obtained using the improved technology.

The temperature–speed conditions of the crystallization process, determined by the rate of movement of the crystallization front (*R*, mm/min) and the temperature gradient at the crystal growth front (*G*, K/mm), significantly influence the intragrain structure, dispersion, and phase composition of the alloy. In Figure 3, the local temperature values for five thermocouples are shown as a function of melting time. The rate of the crystallization front movement was calculated according to Formula (1):

(1) $$R=\frac{∆l}{∆\tau }$$

where ∆l—distance between the thermocouples; ∆τ—time interval for reaching the solidus temperature between the located thermocouples.

Calculations of the temperature gradient at the crystallization front were performed using Formula (2):

(2) $$G=\frac{∆T}{∆\tau \cdot R}$$

where ∆T—temperature interval; ∆τ—time interval; *R*—the rate of crystallization front movement.

The accuracy of temperature measurements was ±5 °C and the distance between thermocouples was 0.1 mm.

**Figure 4:**
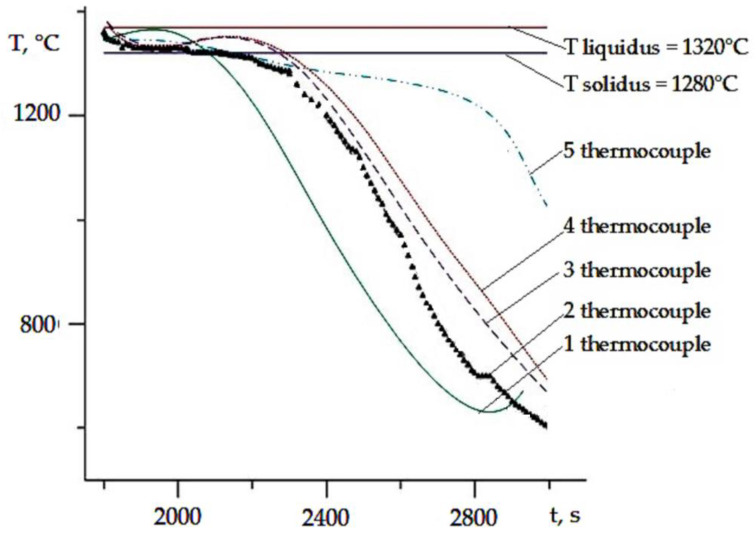
Determination of local temperature values over melting time.

Local temperature values measured by thermocouples 1–5 (Figure 3) were determined as a function of melting time (Figure 4). It was found that the optimal rate of crystallization front movement was 10–12 mm/min, with a temperature gradient on the crystallization front of 165–175 °C/cm. Under these technological parameters of directional crystallization (DC), castings with a regular dendritic structure were obtained.

**Figure 5:**
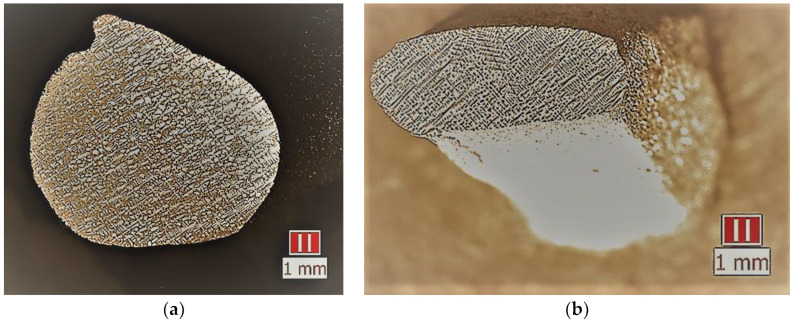
Macrostructure of the sample in the cast state: (**a**) macrostructure of the middle part of the sample; (**b**) cross-section from the seed (near the cone) [2].

Metallographic analysis of the investigated samples of the CM88 corrosion-resistant alloy revealed that, during the study of the macro- and microstructure after the DC process, several types of subgrains could be distinguished. These included subgrains representing individual dendritic branches within one colony, the sizes of which correlated with the sizes of the latter, and the misorientation was 0.5–1.0 degrees. The subgrains belonged to differently oriented groups of dendritic branches with the same direction (Figure 5).

**Figure 6:**
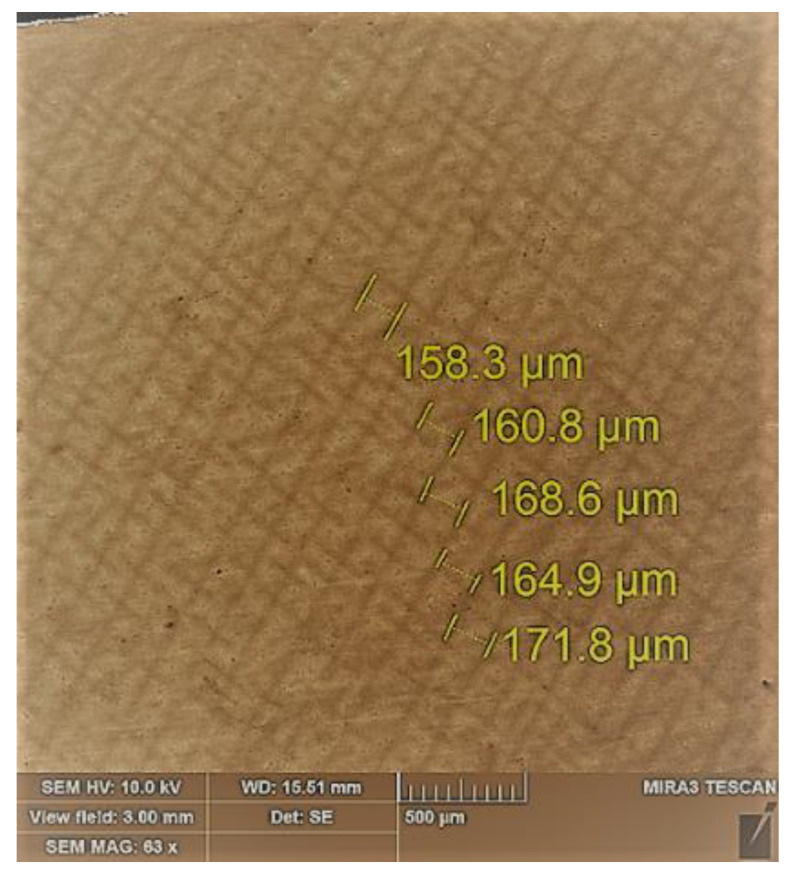
Cross-sectional microstructure of a sample of heat-resistant corrosion-resistant alloy CM88.

**Figure 7:**
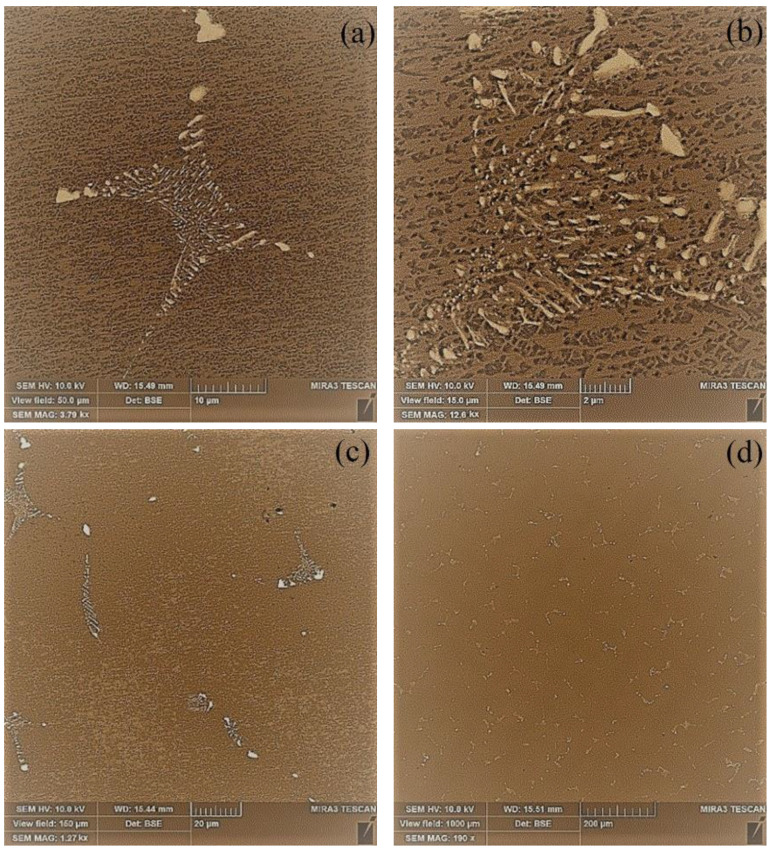
Microstructure of the CM-88 alloy sample at different magnifications (**a**–**d**).

At the speed of the crystallization front in the range of 10–12 mm/min, the macrostructure had a regular pattern, and the degree of regularity of the dendritic structure met the requirements for the structure of the turbine blades of the second stage [36,37]. In the cross-section of the sample, it can be observed that the dendrites have the appearance of a “Maltese cross” [38] with a distance between dendrite axes of this speed λ ≈ 160–170 µm (Figure 6 and Figure 7). The parameters of the structural components of the experimental samples of the heat-resistant alloy CM88 are presented in Table 2.

Casting castings in a VIM-25-175C foundry with additional gas cooling involves an increase in the crystallization rate, but a significant increase in the level of element liquation in the alloy; the appearance of regions of the eutectic high-temperature γ_eut_-phase in the structure was observed (Figure 7a,b). Its volume fraction, determined by statistical metallography for 10 fields of view, was 6–8% at R = 10–12 mm/min. The amount of the secondary dispersed γ′-phase localized in the interdendritic spaces significantly decreased with an increase in the crystallization rate. It was crushed, mainly keeping a shape close to spherical to 0.2–0.3 μm (Figure 7c,d).

Samples for mechanical tests were subjected to heat treatment according to the following regime: homogenization at a temperature of 1170 °C for 2 h; temperature increase to 1180 °C, within 2 h, cooling in air; two-stage aging at a temperature of 1050 °C for 4 h, cooling in air and then at a temperature of 850 °C for 16 h, cooling in air. After heat treatment, the microstructure of the test samples was observed to dissolve the γ_evt_-phase, which had a positive effect on the phase–structural stability of the castings [39,40,41,42,43,44,45,46,47,48,49,50]. The mechanical properties of the samples for short-term and long-term strength met the technical requirements for the CM88 alloy (Table 3). The ultimate tensile strength and time to fracture under long-term load of the test specimens at 900 °C was slightly higher than the regulatory values of 640 MPa and 104 h, respectively, and the relative elongation was 2–3 times higher than the standard requirements [36].

Damage to gas turbine blades is caused by the high temperature of aggressive working gases, stable centrifugal load, and variable loads during turbine shutdown and startup [39,40,41,42,43]. These operating conditions cause corrosion, creep, and fatigue failure of the blades. The resulting CM-88 alloy microstructure (Figure 5, Figure 6 and Figure 7) after DC ensures a high level of thermophysical characteristics, mechanical properties, and microhardness of gas turbine engine blades [39,44,45,46,47,48,49,50,51,52,53].

The perspectives for the development of hydrogen energy [5,16,54,55,56,57,58,59,60,61,62,63,64,65,66,67,68,69,70,71,72,73,74,75,76,77,78,79,80,81,82,83,84,85,86,87,88,89] make it important to assess the resistance of gas turbine materials to hydrogen degradation and the evaluation of modern methods and techniques of their residual lifetime [83,90,91,92,93,94,95,96,97,98,99]. The mechanical properties at short-term tension in gaseous hydrogen at a pressure of 30 MPa of the witness samples made of the studied CM-88 alloy were compared with the properties of the CM-88U-VI alloy produced by traditional technologies [2,33,36,37,40] and the CM-90-VI alloy [2]. The thermal treatment of the CM-88U-VI and CM-90-VI alloys included homogenization for 3 h at 1160 °C, cooling in air, holding for 4 h at 1060 °C, cooling in air, aging at 850 °C for 16 h, and cooling in air. 

The sensitivity of steels and nickel superalloys to hydrogen is determined by grain size and grain boundary structure, the amount, morphology, and distribution of hardening carbides, khalcogenides, and intermetallics, and the tendency of materials to release brittle phases during thermal treatment and high-temperature operation [51,52,53,54,55,56,57,58,59,60,61,67,81,82,83,84,85,86,87,88,89,90,91,92,93,94]. Therefore, the absence of transverse boundaries and finely dispersed allocations of the strengthening γ′-phase provides the high hydrogen resistance of the CM-88 alloy obtained by DC technology (Table 4). Compared to polycrystalline alloy CM-88U, the plasticity characteristics increase; this is especially significant in the CM-88 test samples, both in air and in hydrogen, at 30 MPa (Table 4).

The CM-90-VI alloy was also produced by the method of directional crystallization and has a dendritic microstructure with a thinner γ′ phase in the dendritic zones and a coarser γ′ phase in the interdendritic zones [2]. The values of the strength and plasticity characteristics of the CM-88 alloy and the CM-90-VI alloy are almost identical (Table 4), which indicates the high hydrogen resistance of both materials.

The responsible sourcing of critical raw materials and the sustainable development of renewable energy sources are pivotal in addressing the energy trilemma of ensuring energy security, equity, and environmental sustainability [7]. By leveraging advanced technologies in the manufacturing of hydrogen-resistant gas turbine engine blade alloys, as outlined in our study, we can enhance the efficiency and reliability of energy systems [2,33]. The use of environmentally friendly technologies in the production process, such as water-based binders for ceramic molds [34], not only reduces the environmental impact but also contributes to the sustainable development of the energy sector. Furthermore, the development of hydrogen-resistant materials is crucial for the advancement of hydrogen energy, which is a clean and sustainable energy source [53]. These efforts in material science and engineering are essential for building a resilient and sustainable energy infrastructure, ultimately contributing to the mitigation of the energy trilemma.

The results of the experimental research were transferred to Gas Turbine Research & Production Complex “Zorya”-”Mashproekt”, which manufactures gas turbine engines. In particular:

1. It is recommended to provide a temperature gradient at the crystallization front of ~170 °C/cm to obtain a regular structure with orientation [001] of the cast second-stage turbine blades of the UGT25000 gas turbine engine for power generation.
2. Technological schemes have been developed to obtain ceramic molds made by 3D printing based on polymer models using environmentally friendly binders for the repair of gas turbine engine blades. Their use leads to an increase in the dimensional accuracy of blade castings by 30–45%, significantly reduces the time of casting production, and helps to preserve the health of the company’s employees.

## 4. Conclusions

The single-crystal technological process for the production of blades has improved due to the use of shell molds on a water binder using polymer models. 

We identified polymer materials and a technology of additive manufacturing from them of single-use models for the manufacture of ceramic shell molds, with the aim of creating an ecological technological process and reducing the time of production of high-quality castings.

The use of the VIM-25-175C foundry unit contributed to the intensification of the crystallizer cooling process due to the introduction of an additional inert gas flow cooling unit and guaranteed the achievement of a regular oriented structure for the castings. It was established that a regular directional structure is formed at a speed of movement of the crystallization front in the range of 10 to 12 mm/min, and a temperature gradient at the crystallization front in the range of 165–175 °C/cm.

Tests of witness samples for short-term and long-term strength after heat treatment, according to the standard [36], showed that the level of mechanical characteristics corresponds to that regulated by the technical documentation for this product. Short-term tensile testing of the samples in gaseous hydrogen revealed high hydrogen resistance of the CM-88 alloy produced by the directed crystallization single-crystal technology: the relative elongation in hydrogen at a pressure of 30 MPa increases from 2% for the commercial alloy to 8% for the experimental single-crystal alloy.

## Figures and Tables

**Table 1:** Chemical composition of the investigated nickel-based hydrogen-resistant corrosion-resistant alloy.

Alloy	Content of Elements, wt.% (Ni-Balance)												
	C	Cr	Co	Mo	Ti	Al	W	Nb	Hf	Fe	B	Mn	Si
CM88	0.074	15.17	10.82	1.95	4.40	2.85	5.38	0.18	0.27	4.07	0.01	0.028	0.16
Limits [36]	0.06–0.12	15.0–16.2	10.0–11.5	1.6–2.3	4.2–5.2	2.8–3.3	4.7–5.9	0.1–0.3	0.3–0.6	3.7–4.3	0.01–0.16	≤0.30	≤0.30

**Table 2:** Parameters of the structural components of the experimental samples of the heat-resistant alloy CM88.

Structural Components Parameters	Limits, µm
Average size of eutectic γ′-phase islands	5–7
Average particle size of the secondary γ′-phase in the interaxial space	0.2–0.4
Average size of MC-type carbides	1.0–1.5
The average size of secondary γ′-phase particles in the axes of dendrites	0.1–0.3

Note: The relative accuracy of determining the specified values was 5–10%.

**Table 3:** Mechanical properties of heat-resistant corrosion-resistant alloy at 900 °C.

Sample Number	Short-Term Properties		Long-Term Properties	
	σ_u_, MPa	δ, %	σ, MPa	τ, h
1	650	28	320	110
2	646	23	320	132
3	655	19	320	104
Stardart [36]	640	9	320	104

**Table 4:** Mechanical properties of the alloys in air and hydrogen under a pressure of 30 MPa at room temperature and a tensile rate of 0.1 mm/min.

Material	Test Environment	σ_u_ MPa	σ_y_ MPa	δ %	ψ %
CM-88	air	970	880	12	15
CM-88	hydrogen	950	870	8	12
CM-88U	air	960	890	5	8
CM-88U	hydrogen	930	860	2	4
CM-90	air	970	860	13	16
CM-90	hydrogen	920	820	7	12

## Data Availability

The original contributions presented in the study are included in the article, further inquiries can be directed to the corresponding author.

## Nomenclature and Abbreviations

σ_u_	ultimate tensile strength (UTS)
σ_y_	yield strength (YS)
δ	elongation
ψ	reduction of area
σ	stress during long-term loading
τ	time to fracture under long-term load
LMC	liquid metal cooling
GTE	gas turbine engine
RPM	rotation per minute
GCC	gas cooling casting
HRS	high-rate solidification
DC	directional crystallization (Bridgman–Stockbarger method)
T_S_	solidus temperature
T_L_	liquidus temperature
R	speed of the crystallization front movement
∆l	distance between the thermocouples
∆τ	time interval
G	the temperature gradient at the crystallization front
∆T	temperature interval
λ	distance between dendrite axes
